# Pituitary Adenylate Cyclase-Activating Polypeptide: A Potent Therapeutic Agent in Oxidative Stress

**DOI:** 10.3390/antiox10030354

**Published:** 2021-02-26

**Authors:** Nadia Sadanandan, Blaise Cozene, You Jeong Park, Jeffrey Farooq, Chase Kingsbury, Zhen-Jie Wang, Alexa Moscatello, Madeline Saft, Justin Cho, Bella Gonzales-Portillo, Cesar V. Borlongan

**Affiliations:** Department of Neurosurgery and Brain Repair, University of South Florida Morsani College of Medicine, Tampa, FL 33612, USA; nas146@georgetown.edu (N.S.); bcozene@tulane.edu (B.C.); youjeongpark@usf.edu (Y.J.P.); jfarooq@usf.edu (J.F.); ckingsbury1@usf.edu (C.K.); zwang94@usf.edu (Z.-J.W.); amoscatello@usf.edu (A.M.); saftmad@umich.edu (M.S.); justincho@usf.edu (J.C.); bellagonzales-portillo2024@u.northwestern.edu (B.G.-P.)

**Keywords:** pituitary adenylate cyclase-activating polypeptides, antioxidant, oxidative stress, neurodegeneration, reactive oxygen species, cell signaling, stroke

## Abstract

Stroke is a life-threatening condition that is characterized by secondary cell death processes that occur after the initial disruption of blood flow to the brain. The inability of endogenous repair mechanisms to sufficiently support functional recovery in stroke patients and the inadequate treatment options available are cause for concern. The pathology behind oxidative stress in stroke is of particular interest due to its detrimental effects on the brain. The oxidative stress caused by ischemic stroke overwhelms the neutralization capacity of the body’s endogenous antioxidant system, which leads to an overproduction of reactive oxygen species (ROS) and reactive nitrogen species (RNS) and eventually results in cell death. The overproduction of ROS compromises the functional and structural integrity of brain tissue. Therefore, it is essential to investigate the mechanisms involved in oxidative stress to help obtain adequate treatment options for stroke. Here, we focus on the latest preclinical research that details the mechanisms behind secondary cell death processes that cause many central nervous system (CNS) disorders, as well as research that relates to how the neuroprotective molecular mechanisms of pituitary adenylate cyclase-activating polypeptides (PACAPs) could make these molecules an ideal candidate for the treatment of stroke.

## 1. Introduction

Stroke is a potentially fatal interference in the normal blood supply to the brain that precipitates debilitating sequelae including visual deficits, cognitive decline, and paresis. The two major subtypes of stroke are ischemic and hemorrhagic, although the former is profoundly more common and accounts for nearly 85% of total stroke events worldwide [1]. Ischemic stroke is typically localized to the middle cerebral artery territory that contains critical motor and sensory foci in the brain. The insufficient oxygen delivery results in hypoxia-induced tissue damage and the eventual death of local neurons [2]. Treatment of acute ischemic stroke attempts to restore blood flow to the brain but is limited to the thrombolytic drug tissue plasminogen activator (tPA) [2]. Delivery of intravenous tPA within 4.5 h of stroke reduces mortality and reverses functional deficits. However, failure to administer tPA within this therapeutic window precludes its use, leaving the victim with high risks of developing hemorrhagic transformations and other devastating outcomes [2]. Stroke remains a leading cause of death in the U.S., warranting the need for novel treatments with extended therapeutic windows.

The prolonged perfusion deficit in stroke triggers the ischemic cascade, a three-phase series of pathological biochemical reactions that culminates in cellular death. The cascade begins within hours of flow disruption as the acute phase, which is characterized by ionic disorder, microglial activation, and oxidative stress [3]. The locally hypoxic environment and intracellular calcium surplus promote mitochondrial cytochrome c liberation and subsequent caspase-3 activation that promotes apoptotic cell death [4]. The excess intracellular calcium also stimulates ROS formation via NADPH oxidases that theoretically increases blood–brain barrier (BBB) permeability and advances dysfunction of endothelial tissue [5]. The succeeding steps of the ischemic cascade are the subacute phase, defined by leukocyte invasion and inflammation, and the chronic phase which marks a transition from tissue injury towards limited recovery.

Oxidative stress induces secondary cell death through multiple cell death pathways, including mitochondrial dysfunction [6]. Mitochondria are cytosolic double membrane-bound structures that generate the majority of cellular ATP via the electron transport chain. These powerful subcellular organelles utilize oxygen as the final electron acceptor of NADH and FADH2 to phosphorylate ADP. The newly formed ATP is critical to survival; however, the process of cellular respiration also generates ROS that promote cell death [6]. Normally the mitochondrion detoxifies these free radicals, but in the setting of ischemia-induced damage, mitochondria become dysfunctional or senescent. Once blood flow returns to the site of insult, the sudden surge in oxygen markedly elevates the levels of ROS generated by mitochondria and eventually overwhelms the detoxification capabilities [7]. The oxidative stress from the excess ROS production directly propagates neuronal damage, brain dysfunction, and cell death [7].

Multiple endogenous mechanisms exist to mitigate damage from oxidative stress. For example, creatine kinase expression is upregulated during ischemia and acts as a temporary energy buffer by regenerating ATP from ADP and creatine phosphate [8]. A second mechanism involves mitophagy, which safeguards functional organelles by recycling structural components from damaged mitochondria [9]. Should creatine kinase and mitophagy fail, dysfunctional mitochondria may release cytochrome c into the cytosol to active apoptosis, thus partially circumventing reperfusion-induced oxidative stress [10]. However, the combination of these protective mechanisms is still typically insufficient in combating stroke-induced oxidative stress. Creatine kinase depletes quickly during ischemia, mitophagy is limited by the availability of mitochondria to degrade, and apoptosis leaves lasting functional deficits.

**Figure 1:**
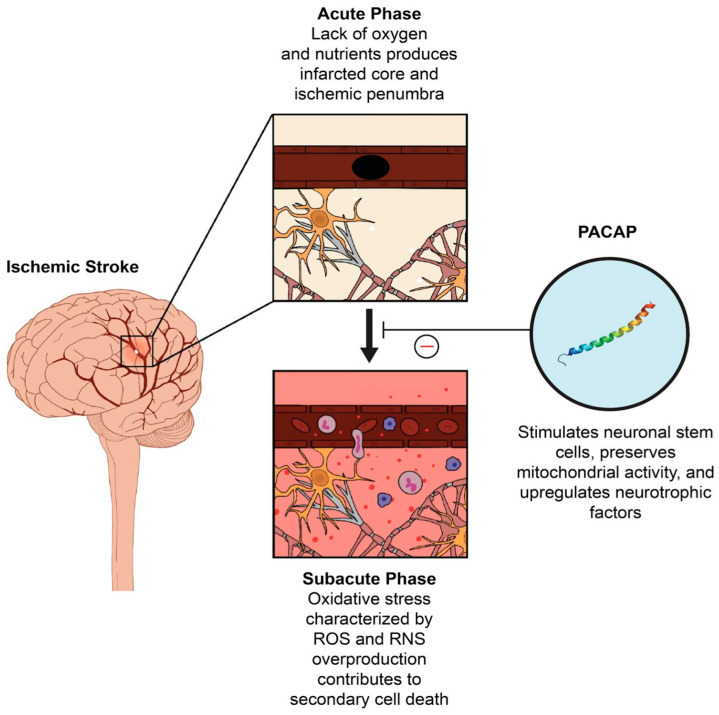
Ischemic stroke entails an acute phase and a subacute phase. In the subacute phase, oxidative stress triggers secondary cell death. Pituitary adenylate cyclase-activating polypeptide (PACAP) may serve as a potent therapeutic intervention in the subacute phase, as it combats oxidative stress and imparts neuroprotection. Reactive oxygen species (ROS) and reactive nitrogen species (RNS).

A less explored endogenous mechanism involves pituitary adenylate cyclase-activating polypeptide (PACAP). PACAP is a neuropeptide that was originally isolated in the hypothalamus but is found extensively in the central nervous system (CNS) and the peripheral nervous system (PNS) [11]. PAC1, the receptor for PACAP, has been identified in the cerebral cortex, brainstem, hypothalamus, hippocampus, and cerebellum [12,13,14]. PACAP is a part of the vasoactive intestinal peptide (VIP)/secretin/glucagon peptide family [15]. PACAP-38 and PACAP-27 are two isomers of PACAP that display neuromodulating and neurotrophic abilities [16]. The majority of PACAP found in the brain is made up by PACAP-38, while PACAP-27 only contributes around 10% of total PACAP composition in the brain [17]. PACAP functions by binding to the PACAP-specific receptor (PAC1R) and can also serve as the agonist for VIP/PACAP receptors (VPAC1R and VPAC2R) [18]. PACAP has been shown to bear neuroprotective properties [11], which may be correlated with the G-protein signaling pathway through adenylate cyclase [19]. PACAP plays a key role in maintaining homeostasis of the CNS and the PNS and regulates the response to injury [12]. It contributes to nervous system development, as it directs proliferation and differentiation of neuronal cells and glial cells [12,20,21,22]. PACAP also functions as a neurotransmitter, potentially mediating cognition and emotion [12,23,24,25,26]. Importantly, PACAP exhibits neuroprotective capacity in stroke and may potently arrest oxidative stress. Therefore, targeted therapeutics focused on upregulating PACAP expression may provide a clinically relevant treatment option for acute ischemic stroke patients (Figure 1).

## 2. The Role of PACAP in Oxidative Stress

Oxidative stress is characterized by an abundance of oxidative radicals, such as ROS, that overwhelm the antioxidative capacity of the organism [26]. Remarkably, PACAP has been shown to act as an antioxidative agent in oxidative stress conditions both in vitro and in vivo. The role of PACAP in the response to oxidative stress was examined by comparing PACAP-deficient mice and wild-type mice [27]. Aged mice with a loss of endogenous PACAP exhibited significantly higher levels of oxidative metabolites in plasma compared to wild-type mice; however, the difference in younger mice was not as substantial [27]. Nonetheless, the findings suggest that PACAP functions as an antioxidant under oxidative stress conditions [27]. Following PACAP38 intravenous treatment, wild-type mice demonstrated downregulated concentrations of oxidative species in plasma and an elevation of anti-oxidative mechanisms [27]. For PACAP-deficient mice, even with PACAP38 administration at a dosage of 5 × 10^−7^ mol/kg, serum levels of oxidative metabolites did not significantly change. Thus, endogenous PACAP plays a critical role in regulating the response to oxidative stress, and a long-term loss of PACAP may culminate in mounting oxidative damage [27]. Additionally, the importance of PACAP for cerebellar development was evaluated by observing cerebellar granule cells harvested from wild type and PACAP-null mice in vitro under neurotoxic conditions [28]. While there was not a significant disparity in cerebellar size or morphology among wild type and PACAP-deficient mice in vivo, cerebellar neurons isolated from PACAP-deficient mice demonstrated increased vulnerability to oxidative stress conditions induced by either ethanol or hydrogen peroxide in vitro [28]. Furthermore, PACAP potentially regulates the response to oxidative stress and confers neuroprotection in hypoxic conditions.

Indeed, preclinical studies have sought to elucidate the mechanisms underlying PACAP’s antioxidative effects. Zebrafish subjected to oxidative stress conditions (hydrogen peroxide exposure) received 100nM PACAP-38 [29]. Hair cells of the inner ear demonstrated decreased caspase-3 concentrations following PACAP-38 treatment. However, levels of p-38 mitogen-activated protein kinases (MAPK), another pro-apoptotic factor, did not change significantly [29]. Inhibited apoptosis on account of reduced caspase-3 levels resulted in ameliorated motor and behavioral deficits spurred by oxidative stress [29]. Moreover, PACAP may mediate the oxidative stress response by suppressing apoptosis, particularly through the attenuation of caspase-3 activity [29]. In ischemic stroke, the build-up of oxidative free radicals, such as, ROS engenders potent oxidative stress, which culminates in significant neuron and glial cell loss [30]. In a culture of cerebellar granule cells treated with hydrogen peroxide, PACAP mitigated cell death, and the extent of amelioration was dose-dependent [30]. The neuroprotective actions of PACAP were inhibited when an antagonist of PACAP, PACAP 6–38, was introduced. Notably, the enhanced cell survival induced by PACAP was curtailed following administration of a MAP-kinase (MEK) inhibitor, indicating that PACAP may mediate neuroprotective mechanisms via MAP-kinase signaling [30]. Additionally, proper astrocyte function is crucial for the response to oxidative stress, as they release antioxidative enzymes superoxide dismutase (SOD) and catalase [31]. However, astrocytes are vulnerable to damage in ischemic conditions [31]. PACAP safeguarded astrocytes from hydrogen peroxide toxicity in vitro, diminishing ROS levels, inhibiting mitochondrial respiratory burst, and suppressing caspase-3 mRNA [31]. Importantly, PACAP reversed hydrogen peroxide-induced inhibition of SOD and catalase, promoting antioxidative activity [31]. Moreover, impaired astrocyte function, an aspect of ischemic stroke pathology, may be amenable to PACAP-based therapy. The mechanism behind PACAP’s antioxidative actions may be the protein kinase A (PKA), protein kinase C (PKC), and MAP-kinase pathways [31]. In sum, preclinical evidence illuminates PACAP’s capacity to combat oxidative stress; however, the mechanisms underlying PACAP’s antioxidative properties warrant further investigation.

## 3. PACAP as A Potential Therapeutic Target in Stroke

Preclinical models have demonstrated the neuroprotective effects of PACAP in ischemic conditions, indicating PACAP’s therapeutic potential. In an in vitro culture of cerebellar granule cells, PACAP promoted viability of neurons and inhibited apoptosis [32,33]. PACAP has also been shown to safeguard rat cortical neurons from glutamate toxicity [34]. In addition, PACAP protected hippocampal neurons from neurotoxicity generated by lipopolysaccharide in vitro [35]. Moreover, PACAP demonstrates neuroprotective capacity, as it suppresses apoptosis and promotes cell viability in neurotoxic conditions. The pathology of global ischemia entails the delayed death of neurons in the hippocampus, specifically the CA1 area [36]. Previous studies have shown that intracerebroventricular or intravenous delivery of PACAP38 attenuates CA1 neuron loss induced by ischemia [36]. PACAP can exert neuroprotection directly via the adenylate cyclase/protein kinase A pathway, but the concentration used in vitro may not be adequate to induce neuroprotection in vivo [36]. Conversely, the therapeutic effects of PACAP may be more indirect, such as upregulating the secretion of neurotrophic elements from astrocytes [37,38,39]. Pretreating neuronal cells with PACAP may protect neuronal cells from ischemic accumulation injuries and neuronal death [40]. Neuronal cells pre-treated with PACAP before oxygen-glucose deprivation and reoxygenation (OGD/R) injury, which mimics acute ischemic stroke injuries, demonstrated upregulated mitochondrial activity and mitochondrial membrane potential in addition to attenuated activity and high mobility group box protein 1 (HMGB1) gene release when compared to untreated cells [41]. Under the same conditions, PACAP pre-treated cells exhibited neuroprotection and reduced neuronal death without hindering primary rat neuronal cell (PRNC) cell growth. PACAP also promoted higher levels of B-cell lymphoma 2 (Bcl-2), brain-derived neurotrophic factor (BDNF), and oxytocin expression prior to OGD, indicating neuroprotective abilities against acute ischemic stroke through neurotrophic factors [42,43,44,45]. BDNF is critical for neuronal survival and plays a pivotal role in cognitive functions [43]; however, BDNF is vulnerable to ischemic injuries. Oxytocin, which is involved in GABA signaling transduction in PRNCs, is necessary to reduce stroke-induced neuroinflammation and oxidative stress [44]. By protecting healthy mitochondrial activity and promoting Bcl-2 and oxytocin expression, post-ischemic injury may be reduced or alleviated.

PACAP’s neuroprotective effects in cell culture have been translated to animal models. In middle cerebral artery occlusion (MCAO) rats, delayed systemic administration of PACAP38 resulted in ameliorated hippocampal neuron impairment following ischemia [36]. PACAP38 treatment resulted in the dilation of arterial blood vessels and an elevation in blood flow to the brain, which may have exerted a positive effect against ischemia [36]. The rats received PACAP38 in the form of a bolus delivered intravenously at a dose of 20 nmol/kg of body weight. A subsequent dosage was provided at 160 pmol/μL per hour over the course of 48 h. At the 4 h time interval, infarct volume decreased by 50.88%. While infarct size did decrease at the 8 and 12 h intervals, the decrease was not as substantial compared to the 4 h period [36]. PACAP38 administration did not show significant adverse outcomes, as mortality was similar among the control and treatment groups [36]. Notably, PACAP’s neuroprotective properties may persist even after its systemic delivery has ceased on account of its ability to bind to the peptide transport system (PTS-6), allowing it to cross the BBB [11]. Because PACAP can easily traverse the BBB, it has potent therapeutic benefits at low doses and does not have deleterious side effects at low concentrations [37]. Furthermore, PACAP exhibits safety and efficacy in mice models of stroke, thereby serving as a potential therapeutic strategy against cerebral ischemia. Importantly, PACAP exhibits therapeutic efficacy over a sustained period of time, indicating a long therapeutic window, which makes PACAP-based treatments a putative therapeutic strategy for stroke. Another in vivo investigation sought to elucidate the role of PACAP in the response to ischemic injury by comparing stroke pathology in PACAP-deficient mice and wild-type mice [11]. Post MCAO, PACAP-38 was delivered intracerebroventricularly or intravenously and culminated in truncated infarct size and improved motor function after 24 h in both experiment groups [11]. The MCAO mice with loss of endogenous PACAP showed an even greater amelioration of neurological damage with exogenous PACAP administration, indicating that PACAP plays a vital role in cerebral recovery from stroke [11]. Evidently, secondary injury following a stroke may be due to changes in transcript expression engendered by cerebral ischemia [11]. Transcriptional alterations post stroke was observed in both PACAP-deficient mice and in wild-type mice that received PACAP [11]. After cerebral ischemia, wild-type mice showed a 50% increase in expression of 228 transcripts [11]. Interestingly, more than half of the altered transcripts have not been shown to be transfigured in stroke [11]. Therefore, treatment with exogenous PACAP generates new gene expression patterns, which may stimulate neuroprotective mechanisms; however, the link between PACAP-mediated neuroprotection and alterations in transcript expression is not yet understood. Since there was a higher increase in transcript expression at 24 h after the initial stroke compared to 1 h post-ischemia, endogenous PACAP may be more heavily involved at later stages of injury [11]. Thus, PACAP seems to play a critical role in the nervous system response to ischemic insult and importantly, demonstrates the capacity to mitigate neurological generated by secondary stroke injury.

While it is clear that PACAP bears neuroprotective effects in ischemic conditions, the mechanism underlying these therapeutic benefits have not been fully elucidated. The following processes mediated by PACAP may contribute to its conferred neuroprotection: hippocampal neurogenesis [46], inhibition of Bcl-2 and caspase-3 [47], promotion of the M2 phenotype of microglia [48], upregulation of BDNF [49], phosphorylation of phosphoinositide 3-kinase (P13-K)/Akt [49] and cAMP response element binding protein (CREB), and interaction with the N-methyl-D-aspartate receptor (NMDAR) [41]. First, hippocampal neurogenesis, particularly in the subgranular zone, is critical for rehabilitation following cerebral ischemia [46]. Immunostaining revealed the expression of PAC1R in adult mice neural stem cells (NSCs), indicating that PACAP is involved with NSC proliferation [46]. Following the delivery of PACAP into the lateral ventricle of the mouse brain, bromodeoxyuridine (BrdU)-positive proliferative cells were upregulated in the subgranular zone, indicating that NSC proliferation increased with PACAP treatment [46]. Moreover, by stimulating neurogenesis in the subgranular zone, PACAP may facilitate post-stroke recovery. Secondly, PACAP protects neurons from damaging ischemic conditions by stifling Bcl-2 and caspase-3 mediated cell death. Bcl-2, the first apoptosis regulator identified, is a potent apoptosis inhibitor that functions by blocking cytochrome c cytoplasmic transportation from the mitochondria, suppressing caspase 9 activation and caspase 3 cleavage [41,44]. In MCAO rats, PACAP38 administration at a picomolar dose attenuated apoptosis via suppression of Bcl-2 and caspase-3 [47]. PACAP38 also exerted neuroprotection by downregulating factors that contribute to neuroinflammation, such as tumor necrosis factor-α mRNAs and interleukin 6 mRNAs [47]. Additionally, PACAP-induced neuroprotection may stem from PACAP’s ability to mediate microglial activity [48]. In vivo, stem cells that secrete PACAP were intracerebroventricularly transplanted following permanent focal ischemia [48]. The upregulation of PACAP levels fostered a steady neurological recuperation [48]. PACAP-induced neuroprotection may in part be due to its ability to transform reactive microglial to the M2 phenotype, which is neuroprotective [48].

In vitro and in vivo studies have shown that PACAP enhances BDNF expression and triggers increased activity of BDNF related pathways, which contribute to neuronal survival [49]. In vitro, cortical neurons subject to OGD underwent PACAP administration. Neuroprotection observed in vitro may have been due to an upregulation of BDNF secretion, which in turn spurs the phosphorylation of tropomyosin-related kinase receptor type B (trkB) and (PI3-K)/Akt [49]. trkB, a BDNF receptor, and PI3-K/Akt have been shown to promote neuronal survival [49]. PACAP treatment also enhanced MAP-2, TuJ1, and GAP-43 expression in cultured cortical neurons [49]. In vivo, PACAP38 exerted neuroprotection against ischemic injury, as indicated by a decrease in infarct volume. Decreased p75^NTR^ and NgR, important suppressors of neuron growth, may have played a role in PACAP-induced neuroprotection [49]. Interestingly, p75^NTR^ functions to mediate trkB activity [50], suggesting that the downregulated p75^NTR^ levels may stem from augmented BDNF. Upregulated BDNF may also activate N-methyl-D-aspartate (NMDA), which then contributes to PACAP-induced neuroprotection [49]. PACAP may also exert neuroprotection via CREB activation. When PACAP binds to PAC1, intracellular cAMP is upregulated and CREB is phosphorylated via protein kinase A (PKA) signaling [51,52]. CREB has been shown to promote cell survival in cerebral ischemia [53,54], and therefore, may contribute to PACAP-mediated neuroprotection.

Another potential mechanism underlying PACAP’s neuroprotective effects is the NMDA pathway. Within the CNS, the glutamate-gated ion channel, NMDR, serves as a regulator of normal physiological function [55]. NMDAR has seven subunits: the GluN1 subunit, four GluN2 subunits (GluN2A, GluN2B, GluN2C, and GluN2D), and two GluN3 subunits (GluN3A and GluN3B) [55]. NMDARs tend to form heterotrimeric groups by either coupling two GluN1 subunits with two identical GluN2 subunits or combining of different GluN2 subunits [55]. Glu2NA and Glu2NB serve as the primary subunits within the CNS, more prominent in the cortex and the hippocampus. Importantly, Glu2NA stimulates survival pathways that are beneficial during ischemic conditions [55]. When extracellular glutamate levels increase, the Glu2NB subunit is activated to mediate cell death [55]. The levels of PACAP are increased in cortical neurons following focal ischemia, due to the presence of NMDAR [56]. The expression of NMDAR is critical for establishing the receptor heterogeneity of the plasma membrane in the neuron [41]. Post ischemic injury, the frequency of inhibitory and excitatory signal transmissions become disproportionate, leading to heightened levels of NMDA-related molecules and restrained NMDAR tracking [41]. Reperfusion following ischemia worsens the insult as oxygen-rich blood circulates to the initial lesion.

During ischemic conditions, neuronal synaptic clefts and extracellular space fill with excess glutamate, inducing neuronal death [57]. NMDA receptors are ionotropic glutamate receptor proteins on the cell membrane that are regulated by Mg2+, GluN2, and membrane depolarization activity. NMDA receptors that contain GluN2B subunits induce apoptosis and reduce CREB pathways via intracellular signaling pathways and caspase 3 activity [58,59]. Certain types of GluN2, namely GluN2C, were found to possess stronger neuroprotective properties against ischemic-induced neuron damage due to their resistance to Mg2^+^ [60]. Additionally, GluN2C expression can be increased via heightened BDNF activity, which rescues vulnerable neurons from NMDAR blockage [60]. In rat models, NMDAR inhibition prevents migraine symptoms from PACAP-38 overexpression and glutamate release, suggesting a correlation between NMDAR and PACAP expression [61]. PACAP, namely PACAP-27, significantly increases GluN2C expression, possibly promoting neuroprotective pathways. Additionally, experimental PACAP-27/38 pretreated cells expressed increased GluN1 and GluN2A activity, correlating to decreased glutamate sensitivity of NMDA [61]. Conversely, lowered GluN2B and GluN2D subunit expressions were observed due to PACAP-induced cytochrome c and caspase 3 cell death pathway regulation, promoting neuroprotective effects. Furthermore, upregulating BDNF expression via PACAP 27/38 pretreatment promotes GluN2C expression [41,62,63], rescuing neurons and enhancing neuroprotection.

While PACAP demonstrates neuroprotective abilities against ischemic stroke injuries via regulation of NMDAR subunits [41], overexpression of PACAP-38 and aberrant glutamate release were seen to induce migraine symptoms in rat models [61]. NMDAR antagonists, such as kynurenic acid and MK-801, can alleviate migraines caused by excess glutamate. However, addiction and other adverse effects arose in rats [64,65]. Due to PACAP’s ability to differentiate [66,67] and proliferate neuronal stem cells [68], balance energy [69], and increase neuroprotection before OGD [41], PACAP therapy for ischemic stroke may induce unique cell proliferation and mitochondrial regulation pathways in neuronal cells with oxidative stress. Further investigations should be conducted to explore the use of PACAP ischemic stroke treatments by targeting NMDAR [68,69].

## 4. Recent Preclinical Evidence Supporting the Therapeutic Potential of PACAP in Stroke

PACAP’s capacity to attenuate oxidative injury, reduce infarct volume, and promote cell survival indicates its potential neuroprotective role in stroke [24,70,71]. The efficacy of exogenous PACAP23 as an agonist for the PAC1 receptor and its neuroprotective effects were examined in neuroblastoma cells [70]. Though PACAP23 demonstrated less efficacy than endogenous PACAP in binding to PAC1, it generated significant therapeutic effects [70]. A One Solution Cell Proliferation (MTS)assay revealed that PACAP23 preserved the functionality of mitochondria and suppressed glutamate excitotoxicity and oxidative injury [70]. Importantly, recent investigations have demonstrated that treatment prior to or following MCAO reduced infarct volume and suppressed neuronal death [18,23,24,48,71,72,73,74,75]. In permanent MCAO mice, those treated with PACAP demonstrated a smaller infarct size and attenuated neuronal apoptosis [18,71]. Evidently, PACAP interacts with several pathways, such as the adenylyl cyclase/cAMP pathway, P12K/Akt, and MEK/ERK, all of which may be involved with PACAP-mediated neuroprotection and anti-apoptotic activity [24]. Moreover, these pathways may serve as potent therapeutic targets for PACAP-driven stroke treatments. Notably, when rats with forebrain ischemia received intravenous and intracerebroventricular treatment of PACAP, cell survival was enhanced in the hippocampus [71,76].

Interestingly, PACAP also plays a role in maintaining the BBB [71,77,78,79]. Receptors for PACAP and vasoactive intestinal peptide (VIP) have been found in the Virchow–Robin spaces (VRS), which are known to play a role in maintaining BBB functionality [77]. Indeed, it has been demonstrated that PACAP has the ability to cross the BBB [71]. Nonetheless, crossing of the BBB can be further bolstered via the use of nanoparticles to deliver therapeutic neuropeptides, such as PACAP [79]. Escalated BBB permeability induced by stroke may spur cerebral edema [78]. In subarachnoid hemorrhage (SAH) induced rats, increased levels of IgG, Evans blue extravasation, and poor cerebrospinal fluid (CSF) circulation were observed, indicating BBB impairment [78]. PACAP expression was abolished in SAH rats, and the rats subsequently received exogenous PACAP [78]. PACAP38 administration reinforced tight junctions in endothelial cells of the BBB, demonstrated by increased ZO-1 and downregulated MMP-9 levels, thereby ameliorating BBB dysfunction [78].

Another mechanism in which PACAP exerts neuroprotection in ischemic stroke entails its capacity to sequester oxidative radicals and promote anti-oxidative processes [80,81,82]. Male Wistar rats received either pituitary adenylate cyclase-activating polypeptide-38 (P38) or an analog of P38 (p38-alg) in an intravenous dose [80]. Treatment with P38 resulted in a decrease in ROS, such as O2- and carbonylated proteins (CP), and an increase in antioxidative enzymes, such as total superoxide dismutase (tSOD) and copper/zinc superoxide dismutase (CuZn-SOD) [80]. With P38-alg treatment, levels of O2- were reduced and tSOD activity was upregulated [80]. While P38 and its analog ameliorated oxidative stress, only P38 significantly upregulated the expression of BDNF [80]. Furthermore, the discrepancies among PAC1 agonists with respect to their anti-oxidative mechanisms warrant deeper preclinical investigation. Notably, in vitro, as H2O2 levels grew, the promoter of the PAC1 receptor was increasingly stimulated in SH-SY5Y cells; however, at very high concentrations, the PAC1 receptor was suppressed [81]. Understanding the hormesis of PAC1 receptor activity under oxidative stress conditions may elucidate a therapeutic strategy in stroke, as preconditioning with low levels of H2O2 may further bolster PAC1 receptor activity [81]. Through the promotion of the endoplasmic reticulum(ER) oxidoreductase and selenoprotein T (SELENOT), PACAP generates anti-oxidative mechanisms [82,83,84]. In sympathoadrenal (SA) precursors, PACAP triggered the cAMP pathway and subsequently activated canonical signaling, which mediated the functionality of mitochondria, leading to transcription of the SELENOT gene [82]. The activation of this gene brought about the antioxidative activity observed during the differentiation of PC12 cells from SA cells. The interaction between the PACAP and the cAMP-mediated AMPK-PGC-1α/NRF-1 pathway induces SELENOT gene expression, which results in the development of high tolerance to oxidative stress [82]. Furthermore, preconditioning with PACAP culminates in greater resistance to hypoxic environments in neuronal tissue, thereby elucidating a potential therapeutic strategy in ischemic stroke.

Along with PACAP’s anti-oxidative properties, PACAP also plays a role in upregulating neurotrophic and anti-inflammatory factors to combat neuroinflammation and neurodegeneration [24,71,83]. At the site of injury in the brain, PACAP stimulates microglial cell activation and astrocyte secretion of neurotrophic elements [83,84,85,86], which aids in alleviating ischemic stroke pathology. During neuronal injury in the peripheral nervous system (PNS), schwann cells secrete neurotrophic elements such as BDNF and PACAP [83]. In addition, PACAP combats neuroinflammation and peripheral nerve deterioration through the suppression of pro-inflammatory cytokines [87,88,89,90]. Evidence has also demonstrated that PACAP in the distal nerve explant upregulates anti-inflammatory factors, IL-4, IL-10, and IL-13 [83,90]. Notably, PACAP may also bear therapeutic potential in stem cell regenerative therapy for stroke, as it stimulates epidermal growth factors and fibroblast growth factors in neural stem cells that enhance proliferation and differentiation [71,86]. PACAP stimulates astrocyte differentiation and proliferation, as well as proliferation of neural stem cells, neuroepithelial cells, and cerebellar granule cells [24]. In sum, PACAP bears rehabilitative potential in ischemic stroke due to its ability to truncate infarct volume, inhibit apoptotic pathways, and recruit anti-oxidative, neurotrophic, and anti-inflammatory factors.

## 5. The Therapeutic Role of PACAP in Non-Stroke Nervous System Disorders

### 5.1. Parkinson’s Disease (PD)

PACAP’s capacity to safeguard neuronal cells from glutamate excitotoxicity and 6-hydroxydopamine neurotoxicity [11,34,81], as well as ameliorate oxidative injury and cell death, elucidates its restorative potential in Parkinson’s Disease (PD) [70,91,92]. The G protein signaling pathways, Gas and Gaq, may play a key role in PACAP-induced cell viability in PD [91,92]. In neuroblastoma cells, PACAP23 promoted cell survival via the activation of G protein pathways, specifically Gαs and Gαq [92]. Another study utilizing neuroblastoma cells demonstrated that pepducins derived from PAC1 imparted neuroprotection against MPP+ induced toxicity, a model for PD [92]. However, only PAC1-Pep1 and PAC1-Pep 2 demonstrated the ability to stimulate Gαq and Gαs pathways [92]. In herbicide paraquat (PQ)-induced PD Drosophila flies, PACAP pre-treatment in the ventral nerve cord (VNS) suppressed caspase signaling and the buildup of ROS, thereby inhibiting neuronal apoptosis [93]. In retinal ganglion cells (RGCs) post optic nerve crush (ONC), PACAP treatment repressed the caspase-3 pathway and upregulated CREB phosphorylation, fostering cell survival [94]. Interestingly, a cationic Argine-rich peptide (CARP), a transactivator of transcription derived peptide (TAT) emulates PACAP and similarly associates with PAC1-R. In vivo and in vitro investigations displayed that TAT acts on PAC1-R alike PACAP, in exerting its neuroprotective effects in a Parkinson’s disease model [92]. Furthermore, TAT was tagged with VIP to facilitate receptor activation, and VIP-TAT exhibited increased efficacy in stimulating PAC1-R when compared to TAT alone [92].

### 5.2. Alzheimer’s Disease (AD)

Alzheimer’s Disease (AD) may be amenable to PACAP-based therapies, as PACAP is involved with learning and memory formation [71,80] and exerts neuroprotection [95]. The capacity for PACAP to cross the BBB was observed in two months old, young, aged, and SAMP8 mice, which is an AD model. It was unanimously shown that PACAP traversed the BBB specifically through the PTS-6 and targeted the hippocampus and hypothalamus [71,96,97,98]. It has previously been demonstrated that one systemic delivery of P38 in rats enhanced memory consolidation [80]. A spatial novelty detection test was employed to observe the influence of intravenous P38 and acetyl-(Ala15, Ala20) PACAP-38-propylamine (ALG), an analog of P38, on memory integration [80]. The P38 rats displayed substantially ameliorated memory synthetization, which was not observed to such an extent with ALG. In the Morris water maze, the p38 group demonstrated more acute searching capacity than the analog group and the control; nevertheless, all three groups successfully completed the task [80]. Furthermore, P38 seems to have a positive effect on memory, indicating its potential therapeutic influence in AD. In the clinical setting, low PACAP concentrations were found in the entorhinal cortex, primary visual cortex, superior frontal gyrus, and the middle temporal gyrus of AD patients [80]. Regarding neuroprotection, a study demonstrated that PACAP administration ameliorated neuronal injury spurred by amyloid beta (Aβ)-stimulated neurotoxicity [95]. In addition, PACAP delivered intranasally upregulated BDNF and Bcl-2 [95]. It has also been indicated that MAPK and P1kK signaling play a role in PACAP-induced neuroprotection in models of AD via α-secretase cleavage of APP, which is a precursor of Aβ [95]. These pathways may serve as a potential therapeutic target for PACAP-based therapeutics in AD.

### 5.3. Amyotrophic Lateral Sclerosis (ALS)

PACAP displays therapeutic potential in ALS due to its neuroprotective and regenerative properties [83,96,97]. In ALS, oxidative stress generates aberrant autophagy, inducing substantial neuronal death [96]. By promoting the mitogen activated protein kinase/extracellular-signal-regulated kinase (MAPK/ERK) pathway, PACAP altered hypoxia-induced autophagy in G93A motor neurons, which led to increased cell viability and inhibited mutant superoxide dismutase (SOD1) aggregation [96]. In addition, when PACAP was eliminated from animal models, the rehabilitation of axons post neuronal damage was significantly curtailed [83]. Evidence suggests that PACAP mediates the release of pro-inflammatory cytokines from Schwann cells and macrophage promotion during nerve degeneration [83]. Importantly, the binding of PACAP to its receptors (e.g., PAC1) leads to the promotion of MAPK/ERK and P12K/Akt signaling, which induces re-myelination [83]. In vitro, motor neurons differentiated from human induced pluripotent stem cells (iPSCs) received neurodegenerative cues. Administration of PACAP protected these cells from cell death [97]. In an in vitro model of amyotrophic lateral sclerosis (ALS), PACAP treatment of motor-neuron like cells mitigated cell death with its mechanism of action being the epidermal growth factor receptor (EGFR) and matrix metallopeptidase-2 (MMP-2) axis [96]. Moreover, the role of MAPK/ERK and EGFR/MMP pathways in PACAP-induced neuroprotection in the context of ALS serves as a crucial source of exploration.

### 5.4. Migraine

Unlike the previous nervous system disorders discussed, PACAP is negatively implicated in migraine pathology [99,100,101]. The manifestation of migraines may be correlated with vasodilation [99]. Vasodilation of blood vessels to the brain has been shown to be augmented in the period of intense migraines, and levels of VIP and nitric oxide (NO) (neurotransmitters involved with vasodilation) are upregulated [99]. Vasoactive neurotransmitters, such as PACAP and calcitonin gene-related peptides (CGRP), may play a role in migraine pathology due to high levels of these neurotransmitters in the trigeminal ganglion. When activated, they induce vasodilation in dural vessels, which can lead to migraine [99]. The dura mater of rats was electrically stimulated to generate a migraine-like headache, and the expression of various peptides of the trigeminovascular system was observed [100]. Following stimulation, levels of CGRP and PACAP were upregulated in the trigeminal ganglion and nucleus caudalis [100]. Notably, it was found that electroacupuncture (EA) treatment significantly decreased concentrations of CGRP, VIP, and PACAP in rat models of recurrent migraines [99]. Clinically, plasma samples from pediatric migraine patients without aura were examined to determine the levels of PACAP and VIP compared to the control [101]. Concentrations of PACAP-38 and VIP were significantly higher in migraine patients than healthy individuals [101].

### 5.5. Neuropsychiatric Disorders

Notable research has also elucidated an association between PACAP and neuropsychiatric illness, particularly stress disorders [24,102]. Subjection to long-term continuous stress culminates in the chronic activation of brain nuclei that impact physiology and the stress response [102], as well as reduced levels of BDNF [102]. Previous studies have indicated that PACAP may be involved with the stress response [102]. In rats with stress disorders, high levels of PACAP and the PAC1 receptor transcripts were found in the stria terminalis (BNST) as well as the hypothalamus [24,103]. In another in vivo study, rats were treated long-term with an agonist of the PAC1 receptor under an environment of stress. When PACAP (6-38), an antagonist of PAC1, was provided, anxiety levels were reduced significantly [24,104]. Interestingly, post-traumatic stress disorder (PTSD) has been correlated with PAC1 receptor methylation, indicating a link between PACAP’s mechanism of action and chronic stress disorders [102]. In the clinical setting, females with PTSD demonstrated upregulated PACAP concentrations in blood, which was associated with greater expression of the PAC1 receptor gene [24,68,105]. Notably, infusion of PACAP (6-38) into the amygdala in patients with intense anxiety and pain ameliorated anxiety symptoms, indicating that PACAP plays a role in anxiety disorder and the pathway serves as a potential therapeutic target [24,106,107].

## 6. PACAP’s Limitations: Barriers to Clinical Use

### 6.1. Potential Side Effects

Although PACAP has demonstrated significant therapeutic promise in preclinical models, it is not yet approved for clinical practice due to side effects, administration route limitations, and metabolic instability. Some of the peripheral effects observed have been cardiac-related complications [108,109,110], cerebral hemodynamics [111,112], and depression [113]. Indeed, PACAP has been implicated in cardiovascular disorders such as chronic heart failure [109] and hypertension [110]. Previously, an examination of human ischemic heart tissue samples revealed increased PACAP27 and PACAP38 immunoreactivity [110], indicating that PACAP may play a role in the pathology of ischemic heart failure. These findings were corroborated in another investigation that evaluated the differences in plasma concentrations of PACAP in patients with chronic heart failure induced by ischemic cardiomyopathy or primary dilated cardiomyopathy [109]. The disparities found among ischemic and non-ischemic chronic heart failure patients indicated that PACAP may contribute to the etiology of ischemic heart failure [109]. Additionally, intrathecal PACAP38 administration may lead to heightened activity of PACAP receptors, which can induce a sustained increase in sympathetic tone, potentially exacerbating hypertension [110]. While intrathecal PACAP-38 delivery spurred prolonged excitation of the sympathetic preganglionic neurons and tachycardia in both hypertensive and control rats, PACAP-38′s impact on mean arterial pressure was inconclusive [110]. While the role of PACAP in cardiovascular maladies, such as heart failure and hypertension, has not yet been fully elucidated, PACAP seems to bear a potent effect on the cardiac system. Therefore, until these effects are understood, PACAP’s use in the clinical context of stroke and neurodegenerative disorders will remain restrained.

Evidence also indicates that PACAP generates neurological and neuropsychiatric side effects, as indicated by its role in cerebral hemodynamics and depression. In an ex vivo study, PACAP38 demonstrated the ability to significantly dilate middle meningeal arteries (MMAs) harvested from rats [112]. Notably, the administration of PACAP (6-38), a PAC1R antagonist, reversed PACAPs effects on the MMAs. Interestingly, PACAP38 had a less significant effect on cerebellar arteries ex vivo. Nonetheless, PACAP38′s capacity to substantially dilate MMAs indicates its potential role in migraine pathophysiology [112]. PACAP27 has been shown to contribute equivalently to migraine pathology compared to PACAP38 [111]. PACAP27′s role in cerebral hemodynamics was evaluated in 18 healthy individuals. In comparison to the control group, those who received PACAP27 infusion demonstrated substantial dilatation of the following arteries: MMA, superficial temporal artery, and external carotid artery [111]. A total of 75% of the subjects who received PACAP27 experienced headaches, while this number was only 17% in the placebo group [111]. Indeed, PACAP seems to play a role in depression, as PACAP-deficient mice have exhibited significant changes in depressive-like habits [113]. When PACAP was delivered intracerebroventricularly to rats, a depressant phenotype was adopted [113]. PACAP administration also resulted in augmented social withdrawal in rats, another aspect of depressive-like behavior [113]. Furthermore, the negative manifestations of PACAP administration in the CNS prevent its clinical approval for a variety of oxidative stress disorders.

Formulating various analogs of PACAP that do not engender these deleterious side effects may serve as a robust area of future research to overcome some of PACAP’s limitations. Notably, Ac-[Phe(pI)(6), Nle(17)]PACAP(1-27) (a compound derived from PACAP) was manufactured and its efficacy was evaluated in vitro and in vivo. Compared to exogenous PACAP, this novel compound showed equal efficacy in protecting SH-SY5Y neuroblastoma cells exposed to MPP(+) neurotoxicity [108]. Importantly, it exhibits greater stability in human plasma compared to PACAP. Regarding side effects, the severity and duration of reduced mean arterial pressure (MAP) was mitigated with the novel compound. In this manner, the safety and efficacy of PACAP analogs should be explored in the preclinical setting in order to translate preclinical findings into clinical practice.

### 6.2. Pharmacokinetic Limitations

Though PACAP has demonstrated neuroprotective effects preclinically, its clinical use in stroke and neurodegenerative disorders is limited on account of its metabolic instability, poor bioavailability, sparse distribution, and quick clearance from the bloodstream akin to other natural peptides [114]. Pharmaceutical modifications of PACAP may serve as a potential solution to overcome the peptide’s poor pharmacokinetic features [115]. Interestingly, chemical alterations of the PACAP, specifically in the *N*-terminus, culminated in increased resistance to degradation by dipeptidyl peptidase IV [115]. In vitro experimentation revealed that PACAP27 exhibits greater stability in human plasma compared to PACAP38 [115]. To bolster the stability of PACAP38, it was chemically transfigured at the endopeptidase and carboxypeptidase cleavage regions [115]. The modifications brought about two novel peptides that demonstrated mitigated metabolic instability and therapeutic efficacy: acetyl-[Ala15, Ala20]PACAP38-propylamine and acetyl-PACAP27-propyl amide [115]. VIP is another peptide similar to PACAP that is also quickly cleared from the bloodstream [116]. A study formulated nine alternative analogs to VIP that display equal affinity to the VIP/pituitary adenylate cyclase-activating polypeptide (VPAC) 1 and VAPC2 but are more metabolically stable than VIP [116]. Among the newly created compounds, 125I-[Ala(2,8,9,16,19,24,25)]VIP seemed especially promising [116]. In sum, the rapid degradation of PACAP and similar natural peptides in the bloodstream creates a significant barrier to utilizing PACAP as a therapeutic in clinical practice. Nonetheless, modifying PACAP to bolster its metabolic stability and bioavailability serves as a potential solution to overcome PACAP’s pharmacokinetic limitations.

### 6.3. Administration Route Problems

Before promising preclinical findings can be translated in clinical trials, the optimal route of delivery for PACAP administration needs to be elucidated. Several routes of delivery for PACAP to the brain have been explored including both local and systematic methods [117]. In terms of local administration, intracerebral, intracerebroventricular, intrathecal, and intravitreal methods have been evaluated. Conversely, systemic routes, such as intravenous, subcutaneous, and intraperitoneal administration, have also been investigated [117]. While the method of intraventricular delivery has been frequently employed in preclinical studies involving PACAP, it may not be the most effective route, as uptake of the therapeutic by the brain from the ventricles is limited [118]. Since the rate of CSF circulation in the ventricles and subarachnoid space is much faster than the diffusion of solutes into the brain, absorption of the therapeutic by the brain occurs at a slower rate than expected [118]. Intrathecal administration of therapeutics seems to be most effective for protein molecules that can withstand degradative enzymatic activity in the cerebrospinal fluid (CSF) [119]. However, intrathecal delivery may leave subjects more vulnerable to side effects in peripheral organs because CSF is reabsorbed by the bloodstream [119]. Another limitation of intrathecal delivery includes the difficulty of determining the optimal dose in order for sufficient levels of the therapeutic to reach the brain from the subarachnoid space of the lumbar region [119]. Similar to the intracerebroventricular and intrathecal routes, intravenous delivery has multiple advantages, but also several limitations that must be overcome. Since PACAP can cross the BBB, intravenous delivery is viable and results in the significant uptake of PACAP by the brain, particularly in the hypothalamus and hippocampus [98]. However, the efficacy of intravenously administered PACAP is limited on account of PACAP’s poor metabolic stability and low bioavailability. In addition, a brain-to-blood efflux transporter for PACAP exists, which restricts the concentration of PACAP that can enter the brain [120]. To resolve these limitations, intranasal delivery has arisen as a potential route. Importantly, intranasal administration would overcome the limitation of poor metabolic stability, as the drug would not have to use blood circulation to access the CNS [120]. Therefore, intranasal injection allows for direct access to the brain and fewer side effects because peripheral organs are not exposed to the therapeutic [120]. However, intranasal delivery does have some limitations. First, the intranasal route may engender a negative reaction in the nasal cavity [121]. Secondly, on account of low surface area of the nasal epithelium, absorption of the therapeutic by the olfactory epithelium and transport to the olfactory bulb is limited [121,122]. Nonetheless, cyclodextrins may serve as a potential tool to bolster the absorption of the peptide-based therapeutics by the nasal epithelium [120,123]. The use of α-cyclodextrin in intranasal delivery of PACAP resulted in enhanced absorption in the olfactory bulb but had the opposite effect in the occipital cortex and striatum [124]. The use of β-Cyclodextrin to bolster uptake of PACAP in the nasal epithelium increased absorption of PACAP in the majority of brain regions besides the striatum and olfactory bulb [124]. Moreover, cyclodextrins should be explored as a therapeutic tool to enhance uptake of PACAP in the nasal epithelium and brain regions. In summary, a multitude of routes for delivery of PACAP to the brain have been evaluated in preclinical models. Means to improve the efficacy of these routes should be explored in future research, particularly intranasal administration, which seems to display the most promise.

## 7. Clinical Trials Supporting Efficacy of PACAP-related Molecules in Stroke and Neurodegenerative Diseases

While the safety and efficacy of PACAP in stroke and neurodegenerative disorders has not been extensively investigated in clinical trials, PACAP-related molecules, such as glucagon-like peptides (GLP-1) and VIP, have been explored. However, the role of PACAP in migraine pathology has been vastly evaluated in the clinical setting. A recent trial among 24 female migraine patients found that PACAP38 administration resulted in a significant increase in migraine attacks [125]. Regarding stroke, an ongoing phase II clinical trial is investigating the efficacy of GLP-1 receptor agonists in subacute stroke [126]. GLP-1 receptor agonists have previously demonstrated neuroprotective properties against oxidative stress in preclinical models. This randomized control study with 30 stroke patients aims to assess the influence of one dose of exenatide, a GLP-1 receptor agonist, on mean flow velocity in the middle cerebral arteries [126]. Encouraging findings from this trial that elucidate the efficacy of exenatide in ameliorating stroke in the subacute phase would suggest PACAP’s therapeutic promise in stroke, as they belong to the same family of peptides. Another ongoing clinical trial is investigating GLP-1 agonists in PD [127]. This study aims to compare concentrations of gut-derived peptides, including GLP-1, in CSF and plasma among PD and control individuals [127]. Additionally, a previous clinical trial explored the role of VIP in AD progression [128]. The brains of 21 AD patients and 46 healthy individuals were evaluated in order to determine any alterations in VIP neurons found in the human suprachiasmatic nucleus (SCN). For female AD patients under 65, the number of VIP neurons in the SCN were significantly reduced [128]. Moreover, VIP expressing neurons seem to be involved with AD progression in human patients, indicating that PACAP may also contribute to AD pathology. All in all, recent and ongoing clinical trials have explored the therapeutic potential of PACAP-related molecules in stroke and neurodegenerative illnesses. Until the limitations of PACAP are overcome preclinically, the safety and efficacy of PACAP in oxidative stress disorders cannot be investigated in the clinical setting.

## 8. Conclusions

Ischemic-reperfusion injury caused by stroke continues to be a major cause of mortality and morbidity in the United States. Ischemic stroke involves insufficient blood perfusion to vital the brain, which leads to oxygen and nutrient deficiency followed by subsequent cell death. The ischemic cascade, prompted by stroke, causes ionic disruption, metabolic failure, increasing permeability of the blood–brain barrier (BBB) leading to an upregulation of inflammation, and mitochondrial dysfunction [5,41,44,45,58,59]. Preclinical studies reveal PACAP’s ability to combat oxidative stress in stroke and other nervous system disorders. Pretreatment of PRNCs with PACAP 27/38 resulted in preserved mitochondrial functionality and upregulated levels of BDNF, as well as suppressed apoptotic signaling [41]. Interestingly, evidence indicates that PACAP-27/38 may coordinate with NMDAR to impart neuroprotection in oxidative stress conditions [41,62,63]. For instance, cells pretreated with PACAP-27/28 demonstrated upregulated GluN1 and GluN2A, which was associated with diminished NMDA glutamate reactivity [41].

Notably, recent research has elucidated PACAP’s role in ameliorating ischemia-induced mitochondrial impairment and glutamate excitotoxicity [92]. Evidence suggests that PACAP also helps maintain the BBB, combats neuroinflammation via secretion of neurotrophic and anti-inflammatory factors, and bears regenerative potential. Some mechanisms potentially underlying PACAP’s anti-oxidative and anti-apoptotic properties include the Gαq and Gαs pathways [70,91], MAPK/ERK signaling [96], and NMDAR [41,62,63], all of which serve as potential targets for PACAP-based therapy. However, further investigation into these pathways is warranted. Additionally, PACAP demonstrates therapeutic promise in a multitude of other CNS disorders, such as Parkinson’s Disease and Alzheimer’s Disease, and chronic stress disorders. New lines of investigation include the role of PACAP in thermogenesis in the regulation of stress response [129]. Beyond the oxidative stress in the central nervous system disorders, a similar stressor is found in peripheral organ perturbations, such as the heart and articular cartilage disease, and PACAP may also play a protective role in the periphery [130,131,132]. Altogether, our understanding of PACAP’s central and peripheral involvement in disease pathology and treatment represents a fertile ground for advancing innovative scientific discoveries and clinical applications. In sum, PACAP was shown to exhibit a rehabilitative role in oxidative stress disorders through its ability to mitigate oxidative injury and hypoxia-induced complications. Moreover, therapies involving PACAP-mediated neuroprotection and the differential action of numerous types of PACP-1 agonists represents a rich area for future stroke research.
